# P-999. Novel diagnostic tools to enhance early detection of congenital Chagas disease

**DOI:** 10.1093/ofid/ofae631.1189

**Published:** 2025-01-29

**Authors:** Melissa Nolan, Stanley Rodriguez Lic, M Katie Lynn, Madeleine M Meyer, Chris Lee, Berry Campbell

**Affiliations:** University of South Carolina, Columbia, South Carolina; University of El Salvador, San Salvador, San Salvador, El Salvador; University of South Carolina, Columbia, South Carolina; University of South Carolina, Columbia, South Carolina; University of South Carolina, Columbia, South Carolina; Prisma Health, Columbia, South Carolina

## Abstract

**Background:**

Chagas disease (CD), *Trypanosoma cruzi* infection, is a neglected vector-borne disease endemic in Latin America for which < 1% of cases have been treated. WHO estimates that ∼9,000 infected neonatal cases occur annually via vertical transmission—US CDC estimates up to ∼300 incident neonatal cases annually. Maternal and neonatal misdiagnosis are principally attributed to the paucity of accurate diagnostic tools for this complex chronic parasitic infection, with current guidelines requiring confirmation testing at >9 months of age—a significant barrier for vulnerable populations already experiencing healthcare access limitations.

**Methods:**

A maternal-infant CD program was established in Sonsonate, El Salvador through a collaboration between flagship universities and the Ministry of Health. Employing machine learning methods, we developed a histology-based parasite image recognition application to aid in triaging of at-risk cases. Second, we developed an innovative diagnostic workflow integrating Bayesian statistics and novel digital PCR methods to validate a new diagnostic tool for enhanced disease detection among mothers-infants.

**Results:**

Since March 2022, a 6% seropositivity was noted among parturition women. Maternal CD status was significantly associated with pregnancy complications and neonatal complications (Apgar < 9 at 1 min and at 5 mins, NICU admission, et al). A customized convolutional neural network design employed on bifurcated Giemsa-stained blood smears revealed >90% parasite detection accuracy. Lastly, McNemar’s statistic found significantly increased diagnostic capacity between our novel dPCR approach vs conventional qPCR. Specifically, dPCR was 2.74 times more likely to detect parasitic DNA in maternal blood than qPCR; 9.5 times more likely in placental tissue; 4.5 times more likely in cord tissue.

**Conclusion:**

Congenital transmission is a growing international clinical concern. Our preliminary results provide compelling evidence for both the need of novel diagnostics and support for incorporation of study-developed cutting-edge diagnostics to aid in timely clinical management of congenital CD.

**Disclosures:**

**All Authors**: No reported disclosures

